# Novel therapeutic strategies and perspectives for metastatic pancreatic cancer: vaccine therapy is more than just a theory

**DOI:** 10.1186/s12935-020-1147-9

**Published:** 2020-03-04

**Authors:** Wenhao Luo, Gang Yang, Wentao Luo, Zhe Cao, Yueze Liu, Jiangdong Qiu, Guangyu Chen, Lei You, Fangyu Zhao, Lianfang Zheng, Taiping Zhang

**Affiliations:** 1 Department of General Surgery, Peking Union Medical College Hospital, Chinese Academy of Medical Sciences and Peking Union Medical College, No. 1 Shuaifuyuan, Wangfujing Street, Beijing, 100730 China; 2 Department of Mechanical Engineering, Tsinghua University, Beijing, 100084 China; 3 Department of Nuclear Medicine, Peking Union Medical College Hospital, Chinese Academy of Medical Sciences and Peking Union Medical College, Beijing, 100730 China; 4 Clinical Immunology Center, Chinese Academy of Medical Sciences and Peking Union Medical College, Beijing, 100730 China

**Keywords:** Vaccination, Pancreatic cancer, Metastasis, Immune therapy, Novel strategies

## Abstract

Pancreatic cancer is an aggressive and malignant tumor with an exceedingly high mortality rate. The quality of life and survival rates of pancreatic cancer patients with metastasis are poor compared with those without metastasis. Thus far, no effective treatment strategy has been established for metastatic pancreatic cancer patients. Therefore, an appropriate therapeutic method based on the elimination of metastatic pancreatic cancer is critical to improve patient outcome. Tumor-targeted vaccines have been widely discussed in recent studies and enabled important breakthroughs in the treatment of pancreatic cancer by preventing the escape of tumor cells from immune surveillance and activating the immune system to eliminate cancer cells. T cells can be activated by the stimulation of tumor-targeted vaccines, but to mount an effective immune response, both immune checkpoint inhibitors and positive costimulatory molecules are required. In this review, we discuss potential tumor-targeted vaccines that can target pancreatic cancer, elaborate the probably appropriate combination of vaccines therapy and evaluate the underlying benefits as well as obstacles in the current therapy for metastatic pancreatic cancer.

## Background

Pancreatic cancer (PC) is an aggressive disease with a poor 5-year survival rate that is mainly attributed to metastasis. PC is often diagnosed at an advanced stage, because the clinical symptoms are not obvious. Chemotherapy is not always successful. Hence, surgery with radical resection is presently the only curative therapy for PC patients. However, less than 20% of PC patients are eligible for operation because of disease progression and metastases [1]. Additionally, because of difficulties in full elimination of PC with surgical resection or chemo-radiotherapy, metastatic PC is currently an unmanageable disease. Therefore, developing novel therapies for metastatic PC is critical.

Immune therapies are classified into active immune such as vaccines therapy and passive immune (or adaptive immune) therapy such as antibodies. Active immune therapies involves a process whereby vaccines target the tumor antigens to enable the patient to mount an immune response and develop immunologic memory. Vaccine-associated immunotherapy is a new treatment strategy in cancer research. Tumor-associated vaccines can inhibit the migration of cancer cells through strengthened immune surveillance. However, the influence of tumor-targeted vaccines on metastasis in PC remains unclear.

This article reviews newly discovered risk factors that are related to metastatic PC along with recent studies on tumor-associated vaccine therapies with the aim of finding more accurate strategies for vaccine therapies towards metastatic PC (Table 1).

**Table 1:** Preclinical and clinical trials of cancer vaccines targeting metastasis PC

Vaccines names	Vaccine types	Targeted disease	Trials	Function	References
OCV-C01	Peptide vaccine	Pancreatic cancer	Multicenter Phase II study	Improve the efficacy of Gemcitabine to PC metastasis	[2]
Ganglioside GD2 targeted vaccine	DC vaccine/Peptide vaccine	Pancreatic cancer	FDA approved	Successfully protect from PC progression	[5]
CA 19-9/KLH vaccine	Conjugate vaccine	Pancreatic cancer	Phase I clinical trials	Successfully protect from PC progression	[8]
MUC1-peptide DC vaccines	DC vaccine/Peptide vaccine	Pancreatic cancer	Phase I pilot trial	Enhance immunological response in metastatic PC	[16]
Synthetic ras peptides	Peptide vaccine	Pancreatic cancer	Pilot I/II study	Enhance immunological response in metastatic PC	[19]
SVN-2B vaccines	Peptide vaccine	Pancreatic cancer	Phase I/II clinical trial	Enhance immunological response in metastatic PC	[22]
Vaccines CRS-207	Whole cell vaccine	Pancreatic cancer	Pre-clinical	Enhance immunological response in metastatic PC	[30]
GVAX vaccination	Whole cell vaccine	Pancreatic cancer	Pre-clinical	Enhance immunological response in metastatic PC	[31]
PAS vaccine	DNA vaccine/Peptide vaccine	Pancreatic cancer	Pre-clinical	Enhance immunological response in metastatic PC	[45]

## Vaccines, tumor-associated antigens and cancer therapy

### Vaccines and PC treatment

Several kinds of cancer vaccines are available, including whole cell vaccines, peptide-based vaccines, dendritic cell (DC) vaccines, DNA vaccines (plasmid vaccines, virus-based vaccines, bacterial vectors as well as yeast-based recombination vaccines) and mRNA vaccines. At present, suppressed and damaged immune system in PC patients are great challenges for cancer vaccines because of the malignancy of cancer, the adverse impacts of chemo- or radio-therapies as well as the advanced stage of PC. However, cancer vaccination involves various strategies to amplify anti-cancer immunity, including the administration of tumor antigens, often with antigen presenting cells (APCs) such as DCs or other immune modulators, or direct modulation of the tumor. Elimination of metastatic PC mainly relies on cytotoxic drugs or cytotoxic immune cells such as CD8+ T cells that kill tumor cells or hinder their proliferation. Nearly all cancer vaccines realize their killing effects by activating tumor-specific CD8+ cytotoxic T cells based on the delivery of MHC class I restricted peptide epitopes derived from shared antigens expressed on the tumor.

In a recent multicenter Phase II study, the peptide cocktail vaccine OCV-C01 combined with gemcitabine (a current first-line chemotherapy) in PC patients (n = 30) showed a median Disease-free survival (DFS) of 15.8 months, which was an improvement compared with gemcitabine alone (a DFS of 12.0 months) [2]. Hence, therapeutic strategies involving the combination of chemotherapy with vaccines may promote the levels of cancer-specific T-cells in immunogenic cancers with stronger outcomes.

### Tumor-associated antigens and PC therapy

Recent studies have shown that PC is an immunogenic tumor and researches on antibodies targeting tumor cells have increased [3]. Antibodies can enhance killing effects of immune-related cells by recognizing tumor-associated antigens (TAAs) expressed on tumor cells [4]. For instance, Dinutuximab, an antibody targeting the TAA ganglioside GD2, has been approved by the FDA [5]. Surprisingly, vaccines targeting TAAs have been reported as potential therapeutic interventions [6]. CA 19-9, also known as Sialyl Lewis, is a carbohydrate TAA that is highly expressed on PC cells [7]. Weitzenfeld et al. used CA 19-9-targeted antibodies produced from the serum of CA 19-9/keyhole limpet hemocyanin (KLH) vaccine-immunized patients to successfully protect mice from PC progression. These results suggest that CA19-9-targeted vaccines could potentially be translated to the clinics [8]. Currently, CA19-9-targeted antibodies are in phase I clinical trials (NCT02672917).

Notably, TAAs may cause serious autoimmune toxicities, as TAAs are expressed in both normal cells and tumor cells [9]. Therefore, antibodies and effector immune cells induced by TAA-targeting vaccines could attack both cancer cells and normal tissues, resulting in increased toxicity. Above all, the identification of a specific antigen that specifically expressed on tumor cells in metastatic PC patients is a novel strategy to precisely target PC cells with reduced toxicity.

### Tumor-specific antigen and PC therapy

In addition to TAAs, cancer vaccines can target other kinds of antigens such as cancer germline antigens, virus-associated antigens, and tumor-specific antigens (TSAs, neoantigens). Tumor-specific antigens are specifically expressed on cancer cells with a low risk of self-tolerance and autoimmunity, which can be successfully targeted by personalized vaccines [10]. TSAs are cancer-specific and patient-specific to induce a stronger T cell response without killing normal tissues. Hence, we speculated that cancer vaccines targeting TSAs can render more immunogenic effects on inhibiting PC metastasis. TSAs can make up for the autoimmune toxicities shortcomings of TAAs. Antigens expressed on PC cells are easily mutated due to their inherent genetic instability. Innumerable non-synonymous mutations have been found in TSAs which makes it become “specific”. TSAs are not expressed on normal cells and thus do not induce autoimmunity. Therefore, targeting TSAs may represent a relatively safe and effective strategy for cancer vaccines compared with targeting TAAs.

Mechanically, TSAs combine with MHC molecules of APCs and are presented by APCs from the cancer vaccine to T cells while the APCs migrating to the lymph nodes. Specific T cells recognize the TSAs to enhance activation of T cell immunity. This activity attracts more specific T cells infiltrating into the tumor microenvironment (TME) to comprise the T cell receptor/MHC/TSA complex, eventually activating the immune response and tumor killing effects of CD4+ T cells and CD8+ T cells. Besides, the destroyed tumor cells can release more TSAs, which not only induce an immune memory but also lead to expansion of T cell activation. Bassani-Sternberg et al. designed a phase 1b trial to determine the feasibility of novel autologous DCs pulsed with personalized TSA peptides in PC patients and found robust CD4+ T cell responses [11]. Therefore, TSA vaccination can generate a highly specific immune response against PC, indicating that a personalized TSA vaccine can bring increased benefits and enhance the function of specific T cells compared with existing immunotherapeutics. In short, we can produce vaccines targeting TAAs as well as TSAs to produce tumor-targeted antibodies, which can inhibit tumor metastasis and progression, while more novel TSAs of PC need to be further explored.

## DC vaccines targeting PC

### How DC vaccines inhibit PC metastasis

**Fig. 1:**
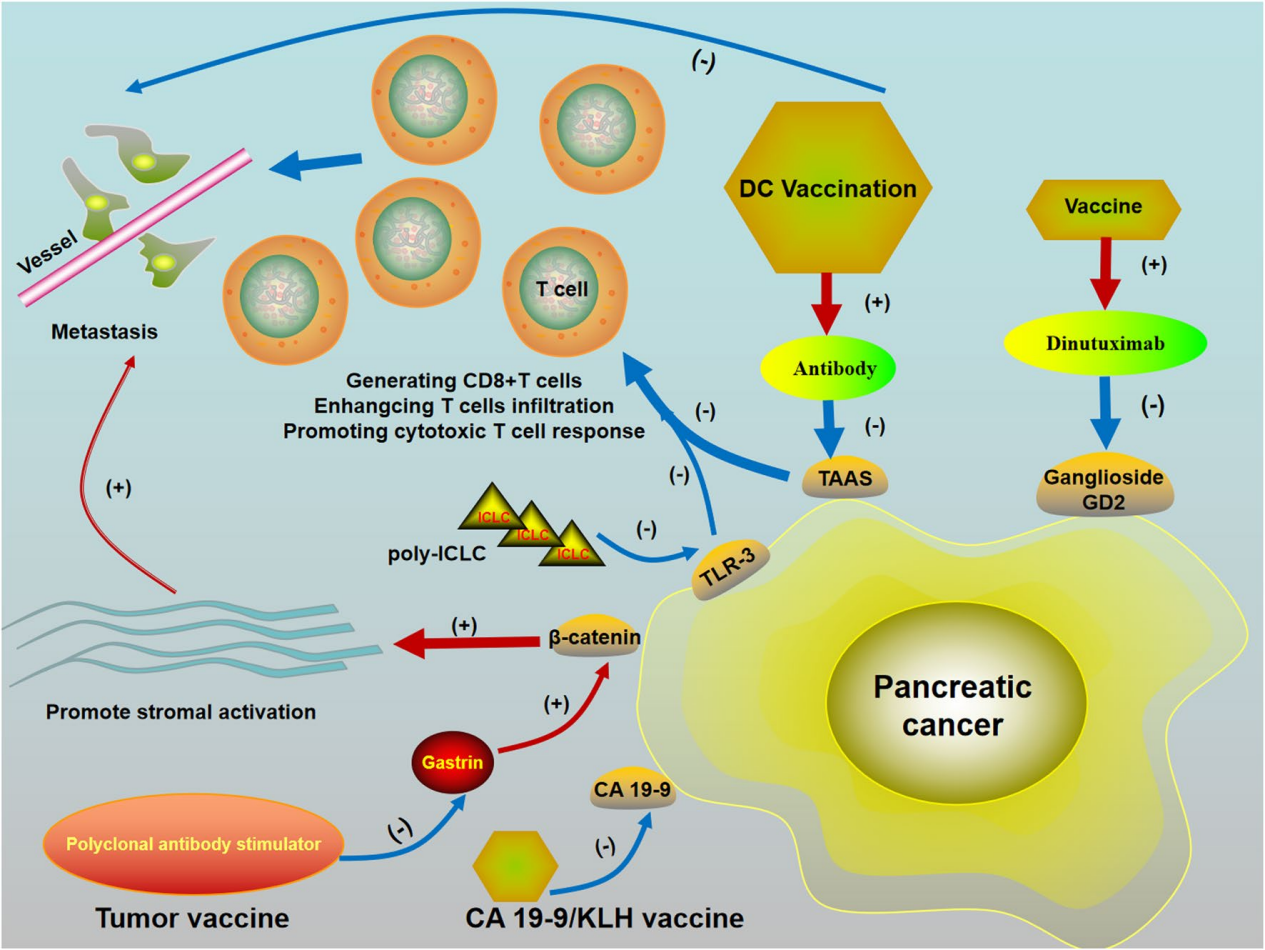
The killing funtion to metastasis pancreatic cancer (PC) of Cytotoxic T cells are promoted by vaccinations. Vaccine therapies that can improve CD8+ T cell ability which are effective in targeting PC metastasis. 1) vaccination with peptide-pulsed dendritic cell (DC) combined with the toll-like receptor (TLR)-3 agonist poly-ICLC is a potential therapeutic method for inhibiting PC metastasis by generating CD8+ T cells; 2) DC vaccines can efficiently enhance cytotoxic T lymphocyte (CTL) response; 3) the tumor vaccine polyclonal antibody stimulator, which selectively targets gastrin, induces system to produce specific polyclonal anti-gastrin antibodies and then decrease PC growth and metastases by downregulating dense desmoplastic fibrosis in the TME, inducing T cell activation. 4) CA 19-9-targeted antibodies produced in CA 19-9/keyhole limpet hemocyanin (KLH) vaccine-immunized patients to successfully inhibit PC progression. (+) Promote (−) Inhibit

Vaccination strategies for PC aim to enhance immune infiltration and endogenous antigen presentation to effector T cells. DC vaccines are a current focus in therapeutic development and can be activated by tumor antigen in treating cancers and as a result promoting cytotoxic T-cell response [12]. DC is a member of the APC family that efficiently present antigens to CD4+ and CD8+ T cells and secrete cytokines such as IL-15, IL-12, IFN-γ and TNF, which promotes activation of cytotoxic CD8+ T cells by transforming the immune response type into a type 1 response [13]. The efficacy of DC vaccination is significantly associated with the amount of DC vaccines migrating to the draining lymph nodes. Large quantities of lymph nodes are present in the abdomen including spleen and peritoneum. Intraperitoneal injection of DC vaccines could enhance the anti-tumor immune response as well as immune memory and inhibit PC metastasis, improving clinical outcomes [14, 15]. Therefore, DC vaccinations may become a potential strategy to inhibit metastasis of PC, and DC vaccination via intraperitoneal injection for immunotherapy in metastatic PC patients may bring about stronger therapeutic responses (Fig. 1).

### Application of DC vaccines in clinics

DC-based vaccines for PC immunotherapy have been proven clinically safe and efficient to induce tumor-specific immune responses. DC vaccination is a potential immune therapy for metastatic PC and the clinical outcomes are often hopeful. Some studies have investigated the potential effects of DC vaccination targeting metastatic PC. For example, Rong et al. conducted a phase I pilot trial on the MUC1-peptide DC vaccine in metastatic PC patients and found the vaccine enhanced the immunological response to the tumor antigen MUC1 in metastatic PC patients without significant toxicity [16]. Mehrotra et al. found that vaccination with peptide-pulsed DCs combined with the toll-like receptor (TLR)-3 agonist poly-ICLC is a potential therapeutic method for inhibiting metastasis of PC by generating CD8+ T cells [17]. Liang et al. examined the cytotoxic T lymphocyte (CTL) responses induced by DC vaccination for PC with the longitudinal assessment of therapeutic responses and demonstrated that DC vaccines can efficiently enhance CTL response and inhibit the migration of PC [15].

## Peptide-based vaccines targeting PC

Peptide vaccines are another important type of cancer vaccine that can induce strong immune therapy by improving stimulation of T-cell immunity. Peptide vaccinations induce numerous antigen-specific anticancer immune responses by recognizing and activating T cells with MHC [18].

Like other vaccines, both specific peptide vaccines and broad peptide vaccines can strengthen effector T cells to eliminate cancer cells, but each has its own strengths and shortcomings. Specific peptide vaccines target TSAs to generate a strong and targeted anticancer immune response with minimal toxicity. Therefore, a special peptide-based vaccine that specifically targets PC should be pursued in future studies. A pilot I/II study by Gjertsen et al. used synthetic RAS peptides as a cancer vaccine in PC patients and found that a strong immune response could be induced [19].

### Peptide-based vaccines and adjuvants

Research has shown the positive efficacy of numerous immunomodulators combined with peptide vaccines. Various strategies have attempted to enhance peptide immunogenicity. Some reports suggested that stronger efficacy can be achieved by co-treating with cytokines or other immune promoters [20]. The reason is that peptide-based cancer vaccines only incorporate MHC-class I-restricted peptides to activate CD8+ T cells, but the incorporation of CD4+ helper T cells activated by other immune agents may also be necessary for PC elimination. For instance, clinical phase I/II trial assessed the efficacy of synthetic mutant RAS peptides in combination with granulocyte–macrophage colony-stimulating factor and found that PC patients showed a stronger immune response to the peptide vaccine with prolonged survival compared with standard therapy (median survival 148 days vs. 61 days, respectively; p = 0.0002) [21]. Hiroaki et al. performed phase I and phase II clinical trials on SVN‐2B vaccines (human leucocyte antigen‐A24‐restricted antigenic peptide) and interferon β for advanced PC. A good immune reaction was demonstrated for the SVN‐2B peptide vaccines with a better objective tumor response rate than placebo, indicating SVN‐2B peptide vaccination could be a potential novel approach for PC and that IFNβ is a good adjuvant for peptide vaccination therapy. Thus, SVN‐2B peptide vaccination with IFNβ adjuvant therapy seems to be an effective and novel strategy for future vaccination research [22].

Altogether, these studies suggest the potential of peptide cancer vaccines in treating metastatic PC depending on effective combination with other immune agents that induce a more comprehensive immune response to PC, indicating that peptide vaccination targeting PC may be safe and may result in a potentially beneficial immune response with co-treatment of an immune adjuvant.

## Vaccine treatments targeting metastatic PC

### Vaccines targeting the TME

TME plays a significant role in metastasis and progression of PC. TME comprises blood vessels and extracellular matrix (ECM), as well as various immune and other cell types. PC chemo-resistance is partly due to the dense fibrotic TME and lack of CD8+ T cells infiltrating. Moreover, cytokines secreted by cells in the TME regulate immune functions, which lead to mass immune responses and promote tumor progression as well as metastasis. Additionally, various components in the TME play both supportive and inhibitory roles in PC metastasis. For example, ECM prevents cancer cell migration but also inhibits T cell attack on PC cells. Various types of vessels transport immune cells to kill PC cells but also provide necessary nutrients to feed PC cells. Therefore, precise and effective strategies to inhibit metastasis by regulating the TME and targeting immune cells in TME are required.

Although vaccines can induce immune cells to attack PC, the TME of PC acts like a barrier that prevents a large number of immune cells from penetrating into it. The TME of PC is characterized by a fibroinflammatory and immunosuppressive stroma. The goal of vaccination is to enhance the ability of tumor-specific T cells and improve direct delivery of immune stimulatory agents into the TME. The key to achieve a positive outcome with vaccination requires both killing tumor cells and the appropriate utilization of adjuvants such as TLR ligands and stimulator of interferon genes (STING) agonists, which can activate DCs and helping DCs infiltrate into TME [23]. Therefore, the combination of vaccine immune therapy and novel therapies targeting the TME could be a promising strategy to improve clinical outcome in PC patients, such as enhancing T cell infiltration by hyaluronidase, strengthening immune cells by IL-2, IL12, as well as reducing the barrier function of TME by TGF-β inhibitor.

Together these studies indicate that a strategy targeting the TME to establish a susceptible environment for vaccine function and tumor-specific T cells may be an effective strategy to inhibit PC metastasis.

## Vaccination and immune cells

### Immune cell phenotypes and PC metastasis

There are various kinds of immune cells associated with tumor metastasis and progression such as monocyte-derived macrophages, tumor-associated macrophages, tumor-associated neutrophils, myeloid-derived suppressor cells (MDSCs), natural killer cell, cytotoxic lymphocyte (CTL) and tumor infiltrating lymphocytes. These cells have capacity to block CD8+ T-cell-mediated tumor killing which as a result lead to the resistance against novel therapies such as checkpoint inhibitor targeting and cancer vaccines [24]. Therefore, understanding the role and function of the immune cells of PC is significant to increase the efficacy of vaccines. Several important immune cells have two phenotypes by producing different cytokines, such as T cells, in which Th1 represents a pro-inflammatory phenotype, and Th2 represents an immunosuppressive phenotype. Monocyte-derived macrophages are another immune cell that has two phenotypes: the pro-inflammatory and cytotoxic M1 type and the immune-suppressive M2 type. Tumor-associated macrophages (TAMs) are associated with both the immune cells and PC cells that promote metastasis and progression of PC and increase chemoresistance of PC [25]. Therapies targeting TAMs in cancer, including Trabectedin [26] and Docetaxel [27], can reduce pro-tumor TAMs and inhibit metastasis.

Tumor-associated neutrophils (TAN) are another type of immune cell in the TME with two types: TAN1 (inflammatory type) and TAN2 (immunosuppressive type) [28]. Furthermore, natural killer T (NKT) cells include NKT1 (anti-tumor) and NKT2 (pro-tumor). Because TAN and NKT play a significant role in regulating immune response, both of TAN and NKT are probably potential targets to inhibit PC metastasis. Myeloid-derived suppressor cells (MDSCs) suppress T cells in the TME, resulting in metastasis. Therefore, inhibition of MDSCs can be a novel therapy to reduce the metastatic potential of PC.

### Vaccination therapies targeting immune cells

**Fig. 2:**
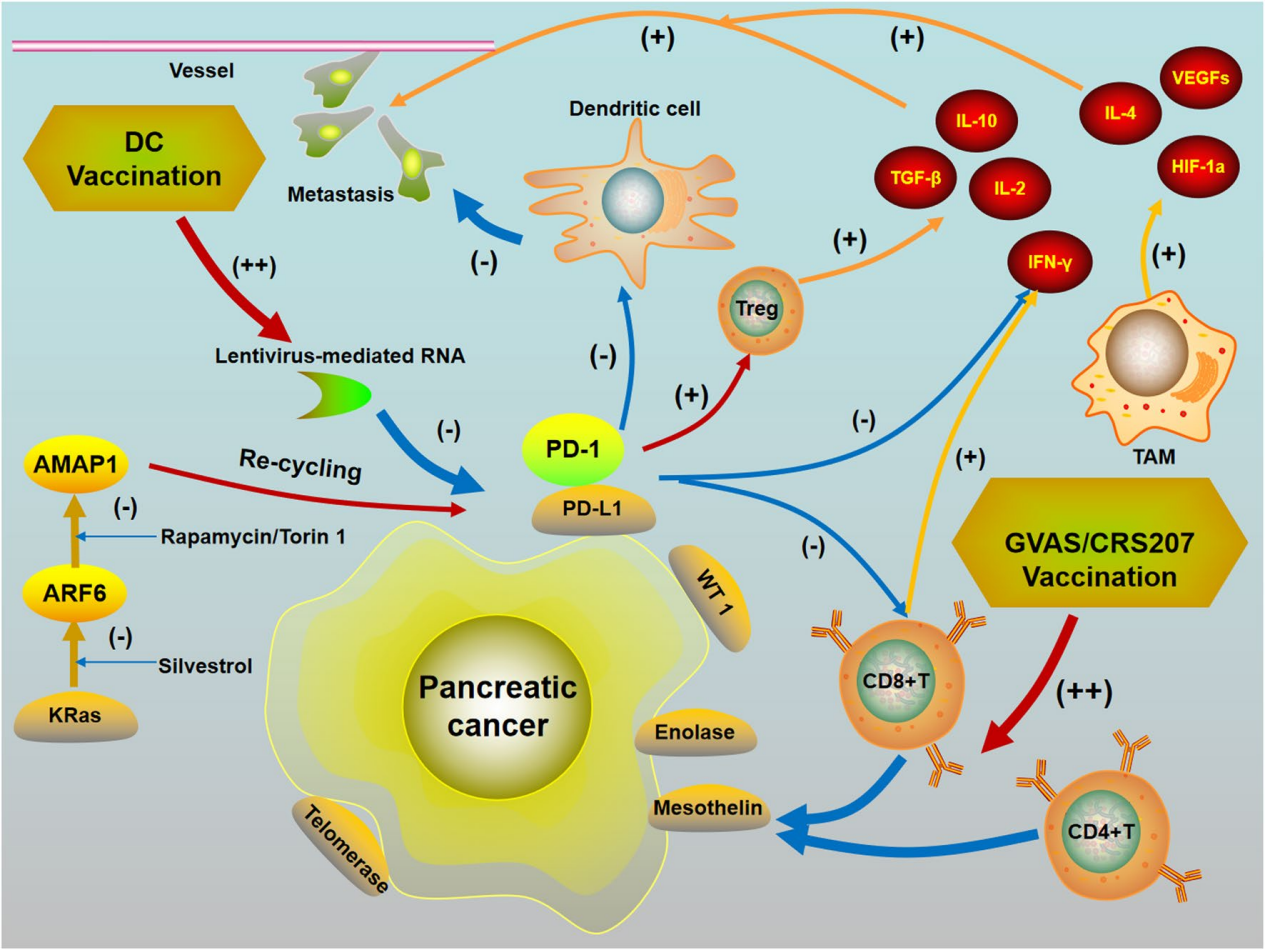
Vaccinations can inhibit the release of various pro-tumor cytokines. Programmed death-1 ligand (PD-L1) inhibits the antitumor effects of Dendritic cell (DC) immunization and regulates regulatory T cells (Treg) which as a result upregulate IL-10 and IL-2 as well as downregulate IFN-γ. 2) ARF6/AMAP1 signaling pathway is closely correlated with the intracellular recycling of PD-L1. Inhibition of ARF6/AMAP1 can reduce PD-L1 cell surface expression. 3) Tumor antigens in PC such as telomerase, enolase, WT1, and mesothelin can stimulate cancer-specific T-cell responses. For example, CD4+ and CD8+ T cells that specifically target mesothelin can be enhanced by vaccination. 4) Vaccines such as live attenuated bacteria expressing mesothelin (CRS-207) and GM-CSF transduced allogeneic whole tumor cell vaccine (GVAX) can improve the immune system in PC by stimulating anti-tumor T cell infiltration. (+) Promote (−) Inhibit

The TME of PC contains various immune suppressive cells that express pro-tumor cytokines. Altering the characteristics of these cells by vaccination may represent an effective immunotherapy strategy. Immune therapies can disrupt the immune-suppressive impact on T-cell function and enhance tumor antigen delivery with vaccines. Tumor antigens in PC such as Kras, telomerase, enolase, WT1, and mesothelin, among others, can stimulate cancer-specific T-cell responses. For example, CD4+ and CD8+ T cells that specifically target mesothelin can be enhanced by vaccination [29]. Vaccines such as live attenuated bacteria expressing mesothelin (CRS-207) can stimulate tumor-specific T cells while inhibiting the function of pro-tumor T cells. GM-CSF transduced allogeneic whole tumor cell vaccine (GVAX) can improve the immune system in PC by stimulating anti-tumor T cell infiltration and myeloid cell activation [30, 31] (Fig. 2).

Together these studies show that vaccination can alter the characteristics of the immune system in TME. Therefore, combination of inhibitors of tumor-suppressing cells with vaccination therapies and increasing protective phenotypes of those immune cells may be a promising strategy.

## Vaccines and immune checkpoints

### PD-L1/PD-1 and vaccines

Programmed death receptor ligand 1 (PD-L1) is overexpressed in many types of malignancies and is a crucial factor in helping tumor cells achieve immune escape. PD-L1 and PD-L2 are ligands for PD-1 that activate immune checkpoints. PD-L1 inhibits the antitumor effects of DC immunization and regulates Tregs and IFN-γ (upregulating IL-10 and IL-2 as well as downregulating IFN-γ), and its expression is statistically correlated with poor outcome in PC patients [32]. ARF6 and AMAP1 are overexpressed in PC and ARF6 activation can down-regulate E-cadherin and upregulate focal adhesion turnover, thus promoting tumor invasion and metastasis [33–35]. KRAS and TP53 oncogenic mutations help activate the ARF6/AMAP1 signaling pathway and both ARF6/AMAP1 and KRAS/TP53 oncogenic mutations were closely correlated with the intracellular recycling of PD-L1. KRAS and TP53 oncoproteins enhance PD-L1 expression via ARF6 and AMAP1, resulting in PC progression and metastasis [36]. Therefore, inhibition of ARF6/AMAP1 or KRAS/TP53 can reduce PD-L1 cell surface expression [36]. The eIF4A inhibitor silvestrol can block ARF6 expression and mTOR inhibitors rapamycin and Torin 1 can inhibit AMAP1. These inhibitors may be novel drugs that can target metastatic PC as immune checkpoint blockades (ICBs) [37, 38].

In PC, blocking PD-1/PD-L1 alone has no significantly positive impact due to the lack of natural infiltration of immune cells such as T cells [39]. Although the potential therapeutic effects of ICB have been demonstrated, most PC patients treated with anti-PD-L1/PD-1 mono-therapy do not obtain satisfying outcomes, with frequent tumor regression or metastasis. Hence, cancer vaccines with ICBs may improve the clinical outcomes of patients with PC by activating T cells and inducing effective anti-tumor immunity because anti PD-1 can increase effector CD8+ T cells and secretion of IFN-γ in the TME, while vaccination could block the immunosuppressive pathways, inhibit immune-inhibiting T cells and reduce CTLA-4 expression on T cells. Bassani-Sternberg et al. hypothesized that vaccination can improve tumor immune recognition in metastatic PC, thus increasing the response to PD-1/PD-L1 blockade [11]. Recently, increasing researches have explored combining inhibitors of PD-1/PD-L1 and cancer vaccines for metastatic PC. Preclinical data indicated that DC vaccines combined with ICBs could generate a synergistic effect with low additional toxicity [40]. Moreover, PD-L1 knockdown by lentivirus-mediated RNA interference combined with DC vaccination may enhance T cell activity in PC on inhibiting metastasis and improving survival [41]. Furthermore, the cancer vaccine GVAX combined with anti PD-1/PD-L1 therapy increased CD8+ T infiltration into pancreatic tumors and improved patient survival [42]. Soares et al. explored the combination of vaccine with PD-1 antibody in an animal study and found that this novel combination can bring much more benefit and improve survival compared with anti-PD-1 alone.

Additionally, gastrin, a gastrointestinal peptide, not only promotes the development of PC but also induce EMT and metastasis of PC by increasing β-catenin [43, 44]. Osborne et al. recently demonstrated that the tumor vaccine polyclonal antibody stimulator (PAS), which selectively targets gastrin, induces system to produce specific polyclonal anti-gastrin antibodies and then decrease PC growth and metastases by downregulating dense desmoplastic fibrosis in the TME, inducing T cell activation, and altering the TME to make it more responsive to immunotherapy with a PD-1 immune checkpoint antibody [45]. We suggest that PAS co-treated with anti-PD-1 immunotherapy may be a novel strategy to inhibit PC metastasis.

Therefore, combining PD-1 or PD-L1 antibody therapy with a vaccine that induces the effects of T cell could represent a novel and efficient strategy for metastatic PC treatment [42]. Thus, a combination of cancer vaccine with these drugs could enhance induction of the immune response and vaccination is thus a potential method to enhance the ability of effector T cells to enter the TME and may play a role in enhancing anti-PD-1 therapy.

### CTLA-4 and vaccines

Another well-known immune checkpoint is cytotoxic T lymphocyte-associated antigen-4 (CTLA-4), which plays a significant role in tumor tolerance [46]. Antibodies targeting CTLA-4 can block the combination of CTLA-4 with its ligands B7 on APCs, which as a result, can inhibit the apoptosis of activated lymphocytes, thereby up-regulating the immune response and producing antitumor activity [47]. Ipilimumab was the first ICB targeting CTLA-4 to be approved by FDA for cancer treatment.

CTLA-4 is mainly expressed on Tregs, which decrease T cell immune responses and help tumor cells realize immune escape. Tregs can express CD25 (the IL-2 receptor), which is vital for their immunosuppressive function [48]. In addition, CTLA-4 inhibits the T cell response by competing for B7 ligands CD80 and CD86. CD80 and CD86 which are expressed on APCs, provide co-stimulatory signals to T cells via CD28. Notably, CD80 and CD86 have a closer affinity to CTLA-4 than the co-receptor CD28. Hence, inhibition of CTLA-4 interaction with CD80 and CD86 can induce CD4 T cell infiltration into tumors. Bengsch et al. showed that antibodies targeting CTLA-4 can stimulate CD4+ T cell infiltration into tumors but cannot enhance CD8+ T cell infiltration into PC tumors. Therefore, other methods such as vaccine therapies that can improve CD8+ T cell ability are likely necessary to strengthen the killing capacity of CTLA-4-targeted antibodies in PC [49]. Thus, cancer vaccines that can stimulate CD8+ T cell activation may be effective in targeting PC metastasis.

Zaidi et al. combined anti-CTLA-4 approaches with an antigen-specific DC vaccine in murine PC models and found that this combination significantly elevated vaccine-induced CD4+ T cells as well as CD8+ T cells in PC, providing a novel idea of the combination of vaccine therapy with ICB to drive T cell infiltration in murine PC models [50]. Hence, the combination of ICB, immune-enhancers and vaccination represents a potent therapeutic strategy to target PC metastasis.

In conclusion, recent developments in chemotherapy have improved the overall survival in PC patients, but the mortality rates remain high. The use of multiple vaccines has become more prominent due to demonstrated immunogenic and safe outcomes, but the application of vaccination alone can induce immunosuppressive Tregs and MDSCs. Above all, ICBs such as anti-PD-1 and anti-CTLA-4 have improved outcome in PC patients. The combination of cancer vaccines with checkpoint blockade (anti-CTLA-4, anti-PD-1) might strengthen the antitumor effects with stronger immune response but lower toxicity.

## Boosting vaccines with adjuvants

To overcome the immunosuppressive characteristic of PC and lack of enough specific antigens, adjuvants are needed for PC immunotherapy. Current literature has demonstrated the potency of cancer vaccines in combination with adjuvants to obtain efficient immune responses and delivery in metastatic PC. Adjuvants have little ability to eliminate PC cells but can promote the immunogenicity of vaccination.

GM-CSF is an important cytokine that can promote the antigen-presenting ability of DCs. GVAX is a whole cell-based vaccine that can express GM-CSF. As an adjuvant, Cyclophosphamide(Cy) can inhibit the immune-suppressive Tregs, and GVAX vaccination combined with Cy improved the overall survival rate of metastatic PC patients compared with GVAX vaccination alone(130 vs. 69 days) [51].

Additionally, GVAX combined with Listeria monocytogenes (Lm)-based vaccine has shown positive outcomes of inducing stronger DC function and activating elevated cellular immunity. CRS-207 is a live-attenuated strain of Lm engineered to express mesothelin, a TAA of PC. Le et al. demonstrated that CRS-207 boosted the clinical outcomes of Cy/GVAX treatment and Cy/GVAX followed by CRS-207 significantly improved overall survival compared with Cy/GVAX alone in metastatic PC patients [29]. Based on the observed survival and favorable safety profile, Cy/GVAX and CRS-207 are being explored as a treatment for PC.

Besides, CD40, a surface member of the TNF receptor superfamily, can strengthen vaccine effects and antigen-specific CD8+ T cells, dependent on the increased IL-12 (critical T cell stimulatory cytokines) secreted by CD40-expressing DCs [52].

Furthermore, Blair et al. showed that indoleamine-2,3-dioxygenase (IDO1) which is expressed on PC, can induce the resistance to immunotherapy. GVAX vaccine can induce IDO1 expression but IDO1 overexpression is closely associated with poor survival following GVAX treatment. Thus an IDO1 inhibitor (EOS200271) enhanced antitumor efficacy of GVAX by increasing T cell amount and function in PC. These results indicate that the combination of vaccine and IDO1 inhibitor could be a novel and effective therapeutic strategy for PC metastasis and suggest the importance of inhibiting IDO1 in vaccine therapy [53].

To make the best use of adjuvant on vaccines, nanoparticles or microparticles have been found as carriers for co-delivering vaccine adjuvant and antigen and protecting vaccine components from hydrolyzing, which can enhance immunogenicity while maintaining their original efficacy [54]. Particles containing both antigen and adjuvant showed better outcomes than antigen only. Particles combine particulate cancer vaccines and other immunomodulation reagents will become a novel strategy in PC immunotherapy.

In conclusion, vaccines targeting tumors have shown great promise in the safety and feasibility in metastatic PC treatment. To strongly enhance immune response, cancer adjuvants should be co-administered with vaccines to achieve more effective delivery in TME, as adjuvants can enhance the immunogenicity of the vaccine. We suggest that the combination immuno-therapies and vaccines are effective approaches in cancer immunotherapy to decrease immunosuppression and augment anticancer effects.

## Conclusion

In this review, numerous underlying mechanisms of vaccines and novel therapies for metastatic PC have been discussed. Immune system response to TME play a dominant role in affecting the metastatic capacity of PC cells. Vaccines that induce a T-cell response may enhance the efficacy of immunotherapy. Therefore, the combination of vaccine therapies with drugs targeting the TME to enhance the infiltration of T cells can strengthen the effect of inhibiting PC metastasis. Besides, we highlight the importance of inhibiting immune checkpoints in the application of vaccine therapy. This approach may contribute to further optimization of the best combination of drugs and vaccines for metastatic PC patients.

## Data Availability

No additional data available.
